# Home Libraries in Boston

**Published:** 1892-04-16

**Authors:** 


### Home Libraries in Boston.

We doubt whether young Jonathan is by nature a book-lover. From what we know of him, we should say that his tendency is to be up and doing, rather than reading or dreaming; and that if we must acquiesce in the barking and biting of our canine friends on the ground that "'tis their nature to," we must for the same reason acquiesce in the American youngster's habit of poking his nose into his elders' business affairs, love affairs, and what not, and airing his knowledge in a perky, irreverent manner which, if he had lived in the days of our great-grandmothers, would have resulted in his being promptly whipped and sent to bed. Still, he has his times of quiet, and very important it is to cater for those times—to give him something to read which shall keep that active little brain out of mischief, or, better still, provide it with something on which it may profitably work.

It is doubtless true that there is an enormous mass of literature in the New as in the Old World—enough, one might think, for all and sundry, old and young. But it will by no means do to leave children without guidance and support in this matter—to give them no encouragement to read good books, and allow them to pick up their reading as the birds pick up food, be it in the sweet, open fields, or in the gutter. We do not, indeed, believe that indiscriminate reading is so universally harmful as some would make out; certainly, we have ourselves known cases in which a child has been allowed to browse at will in a general library, and has carried away nothing but good, simply letting slip all to which its innocent little mind gave it no clue, and finding out with amazement in after-life that it had read books which were considered decidedly "naughty." To the pure all things are pure; there is a deeper truth in that saying than those who for ever harp upon the natural depravity of the human heart can readily understand.

At the same time, we must remember that the converse of the proposition is also true. A mind predisposed to evil will find it in many places where the pure mind may rove unharmed; and even an insatiable curiosity, such as many children are born with—and American children, we fancy, more often than their English cousins—will lead to much mischief being done, if its little possessor is left without guidance and restraint. Moreover, it is a lamentable fact that the children of our lower classes, let them live in what country they may, must of sad necessity become acquainted with many forms of evil of which our own little ones, in their carefully guarded homes, never so much as dream. It follows as a natural consequence that if bad books fall in their way, they can readily interpret much that would be simply unintelligible to a middle or upper class child; and, apart from this, is it not of the highest importance, seeing that the lives of these poor little children are what they are, to take care that there shall be a pure, wholesome-aired world—a world, that is, of books—into which they can sometimes step, a world in which there are sunshine and flowers, loving parents, happy children, plenty of fun, but no sin—or where, if sin there be, it is called by its proper name, and given its proper punishment? Not that the books should be "goody-goody"; indeed, that is just what they should not be, if they are to be really useful. But our American friends, we think, have a keen sense of the difference between good and goody, and act upon it. Some of the books used by the society whose work we are about to describe, being by American authors, are naturally not well known to us; but many of them we do know, while others, again, are by English authors; and we have no hesitation in saying that librarians who choose out books by such writers as Louisa Alcott, Mrs. Molesworth, and Mrs. Ewing, are in no danger of setting namby-pamby fare before their little patrons.

But to come, without further words, to the work of the society with which we are concerned. Four years ago, the Boston Children's Aid Society, having doubtless before its mind some such considerations as those we have just been urging, resolved to start a "Home Libraries" branch, which (we quote from the report) "places in the living-room of some poor family, where there is a responsible boy or girl of thirteen or fourteen who can act as librarian, a small bookcase, with a glass door and a lock and key, the case being screwed to the wall. In this case are placed fifteen books and five periodicals, each library set being composed of books which have been carefully read by the Committee (special care being taken that there shall be nothing in them which could possibly be offensive to any religious denomination), fairy tales, stories of home-life, travels, and the lighter kinds of history. The 'Home Library' having been duly fixed and stocked, ten children are allowed to join the 'group,' free of charge, not more than two in a family being admitted. The books, however, may be used by others than the regular members; and in many cases the parents read them as freely as do the children. In three months a library is generally pretty nearly read through, and it then passes on to another 'group' of readers, being replaced by a fresh one."

For four years now the Home Libraries have been at work, and forty-six have now been opened in Boston, the number of members being about 460. There is a demand for many more, and the Committee much hope to start thirty more during the present year, if funds will permit. The initial cost of establishing a new library they find to be about twenty-five dollars—say, five pounds.

But a special feature of the work yet remains to be noticed. While the librarian is one of the children themselves, each library has a "visitor"—a lady or gentleman who undertakes to meet the children once a week, when the books are exchanged, and to talk over the books with them, encouraging them to read thoroughly, and to "glean from the books the points best worth remembering." As is only natural, a strong and wholesome friendship springs up between the visitor and the children; and plans are being made for the transference of the children, as they grow up, into an Adult Readers' branch, where the visitor will still have supervision over the books read. Whether these books will then be provided by special libraries, or whether the young readers will be expected to obtain them from the public libraries, we are not quite clear.

It is indisputable, we think, that such libraries must play a very useful part in the education of the children of the Boston poor. The Committee report that "interest in reading the best books is developed; the children's pride is stimulated by a sense of proprietorship and organisation; home amusement and occupation become realities; the family tie is strengthened; and individual character developed."

And if it be argued by some that the work could be equally well done for hundreds at a time instead of for tens and without all this paraphernalia of home libraries, groups, and visitors, we would answer, Not so. Centralisation is a curse and not a blessing if it substitute dealing with people in a lump for personal influence. To prevent overlapping, and to ensure that the work shall be thoroughly done, we must have centralisation; but we must also have something much higher; and to our thinking these home libraries present a happy combination of the two. We should much like to see the scheme tried in England. But failing this, why should not managers of parish libraries make something more of the weekly meeting than a formal exchanging of books and putting down of names—encouraging the children to talk of the books they have read, learning what they like and why they like it, seeking to establish, over the books, a friendship which may influence the whole after-life of these little readers? It is a chance which is not to be despised, an opportunity which ought not to be neglected.

